# Diversified dietary intake and associated factors among pregnant mothers attending antenatal care follow-up in public health facilities of Dire Dawa, Eastern Ethiopia

**DOI:** 10.1371/journal.pgph.0000002

**Published:** 2022-06-30

**Authors:** Efrata Nigussie, Abebe Ferede, Melese Markos

**Affiliations:** 1 Public Health Expert at Ohio State Global One Health initiative, Dire Dawa City Administration, Dire Dawa, Ethiopia; 2 Department of Public Health, School of Health Science, Arsi University, Asella, Ethiopia; 3 Department of Public Health, College of Health and Medical Science, Wachemo University Durame Campus, Durame, Ethiopia; The University of Texas Health Science Center at Houston School of Public Health, UNITED STATES

## Abstract

Poor diversity dietary intake has great significance to women, pregnancy outcome and on her fetus or the growing and development of their baby collectively. Uncertainty of studies to examine whether pregnant women have been utilizing diversity dietary in their frequent intake and this has changed nutritional status. Therefore, this study aims to assess diversified dietary intake and associated factors among pregnant mothers attending antenatal care follow-up in public health facilities of Dire Dawa, Eastern Ethiopia. A facility-based cross-sectional study was conducted among 453 pregnant mothers randomly selected from the antenatal care unit at public health facilities from November 1-30/2020. Study subjects were selected with a systematic random sampling method from randomly selected antenatal care unity of health facilities in Dire Dawa Administration. A structured questionnaire and anthropometric measurements were used to collect data. Data were entered with kobo software and exported to Statistical Package for Social Science statistical software version 20 for analysis. Binary and multiple logistic regression models were used to declare the significance of independent variables at P<0.05. This study shows 55% (95% CI = (50–59.5) were inadequate diversified dietary intake. Having lower monthly income (Adjusted Odds Raito [AOR] = 4.4, Confident interval [95%CI] = 1.3–14.6), elementary educational status of mothers (AOR = 3.8, 95%CI = 1.5–9.9), consumption of two meals per day (AOR = 16.6, 95% CI = 2.04–135.8), didn’t get antenatal care counseling (AOR = 2.2, 95% CI = 1.1–2.8) were significantly associated with diversified dietary. The result revealed that around 55% of respondents had inadequate dietary diversity. low household income, had less maternal education level and, consuming two meals per day, and no information about dietary diversity has contributed to inadequate dietary diversity. Accordingly, go forward in the right side of those variables were the core recommendation of this study.

## Background

Dietary Diversity is the feeding of an adequate variety of food groups. Sufficient maternal nutrition throughout the “first 1,000 days” window is mostly critical throughout a woman’s pregnancy through to the child’s second birthday. Improving the nutritional status of the woman before and during pregnancy can reduce the risk of adverse birth outcomes, such as low birth weight and pre-term birth [1–3].

A Diversified Dietary is one of the greatest strategies extremely suggested among pregnant women, which is associated with enriched diet competence through improved food groups in daily diet. Dietary diversity takes been defined as the number of diverse food groups that are consumed over a definite reference period [4–6]. Good maternal nutrition is significant to health and growth. Nutrition throughout pregnancy is different from the non-pregnant state; sufficient dietary feeding during pregnancy is needed to suitable birth outcomes and good health for the mother [7–10].

Pregnancy requests a good diet that contains a sufficient intake of energy, protein, vitamins, and minerals to meet maternal and fetal desires. In Poor countries in sub-Saharan Africa, south-central, and Southeast Asia, maternal undernutrition is highly prevalent and has poor perinatal outcomes [11].

A pregnant woman with advanced dietary diversity makes definite the adequacy of dietary diversity for their children and families. Inadequate dietary diversity at the individual, household, and community levels, or any combination of these, may be influence factors to poor nutrition status which is an intergenerational cycle of malnutrition [12]. Day-to-day calorie consumption among pregnant women is made-up to be about 1,800 calories per day during the first trimester, 2,200 calories per day during the second trimester, and 2,400 calories per day during the third trimester [13].

Globally, around 2 billion people, most women and young children, are affected, by micronutrient deficiencies, with even higher rates during pregnancy [14]. Nearly all (99%) of maternal deaths annually occur in developing countries [15].

About 870 million people are probable to be underweight around the world. Out of these, 852 million were in developing countries [16]. In further, 3.5 million women and children aged under five in developing countries die each year due to the underlying cause of undernutrition. Around 800 pregnant women die every day during pregnancy and childbirth and 8,000 newborn babes die within the first month of life in developing countries [12]. Inadequate dietary intake may lead to low birth weight, stillbirth, the premature rupture of membrane, intrauterine growth restriction, intrauterine fetal death, and congenital anomalies and affect later on sudden infant death syndrome, developmental impairment, malnutrition, and threat for chronic disease [17–19]. Universally, mortality and morbidity due to undernourishment have not meaningfully changed over the last 30 years, to advance it, the dietary diversification status of pregnant women needs to sustainably improve through addressing evidence and reduction of obstacles of obedience [20].

Poor diversified dietary consumption during pregnancy is an important contributor to international maternal malnutrition in less developed countries. A previous review showed that pregnant women in developing countries suffer from energy insufficiencies due to relatively insufficient energy intake. Besides, 42% of pregnant mothers globally and more than 50% of pregnant mothers in developing countries are anemic, mainly due to iron deficiency [21, 22].

In Africa alone, 20% of women are underweight, Even however dietary diversification is significant for the health of the mother and the fetus in Ethiopia, 22% of women are thin due to inadequate diet diversity (vegetables and fruits), and fluctuating regimes [23]. Still, a high problem of maternal malnutrition due to diversified diets are mostly based on starchy foods with slight or no animal products and few fresh fruits and vegetables [24]. Studies were done in different parts of the Africa region, Kenya and Ghana shows that 43.9% and 74.5% of women get minimum dietary diversity score (MDDS) respectively, and also mean dietary iron intake from 3.8 to 97.8 mg/d and 34–100% of the women’s reproductive age (WRA) in Kenya, Nigeria, and South Africa had inadequate intakes) [25–27].

In Ethiopia, Iron consumption is described to be high (47–97.8 mg/d) and only 8–12% of the women’s reproductive age (WRA) had inadequate intake. Mean dietary vitamin A intake reached from 71 to 2477 μg/d, and 3–100% had inadequate intake. Mean dietary zinc intake from 3.8–16.2 mg/d and 23–96% had inadequate intake in Ethiopia [27]. Organs are being designed, and the fetus develops at an enormously rapid rate, this all leads to improved nutrient needs that want the pregnant women to grow both the diversity and the total of foods consumed [28]. Ethiopia’s administration launched the National Nutrition Program and prioritized interventions like, Stimulate maternal nutrition containing adequate intake of diversified foods to develop the nutritional status of women. The application of the above strategy, thinness, and different micronutrient deficiencies are common problems during pregnancy [23, 29].

A cross-sectional study was done in Dire Dawa 2017, Dietary diversity and nutritional status of pregnant women attending a public hospital, high undernourished and low diversified dietary of pregnant women were found [30]. However, there is little known about the relationship between dietary diversity and associated factors among pregnant mothers in Eastern Ethiopia, particularly this study assesses the impact of health service utilization, maternal illness, and meal frequency on diversified dietary intake of pregnant mothers. Therefore, this study aims to assess dietary diversity and associated factors among pregnant mothers both in urban and rural public health facilities in Dire Dawa.

## Methods and materials

### Study setting, design, and population

The facility-based cross-sectional study design was conducted in Dire Dawa city administration from November 1-30/2020. Dire Dawa is one the administrative city, which is 515 km far from Addis Ababa, a capital city. Dire Dawa’s 2020 population is now estimated at 408,096 and the annual growth of the population was 4.37%. There are two governmental hospitals, four private hospitals, five higher clinics, twelve medium clinics (Private), fifteen health centers, and thirty-four health posts with 100% health service coverage. All public health facilities give Antenatal care. The total number of pregnant mothers come to public health on ANC Follow up in a single month 1256.

### The population of the study

All pregnant mothers attending antenatal care at public health facilities of Dire Dawa from 12 weeks gestational age, were the source population and those pregnant mothers who were selected among Antenatal care follow-up from selected public health facilities during the data collection time were the study population.

All pregnant mothers attending antenatal care at the selected facility were included in this study.

During the collecting time, the pregnant mother’s ill and/or difficulty communicating was excluded.

### Sample size determination

The required sample size was determined by using a single population proportion formula with the following assumptions: $n=\frac{z^{2}pq}{d^{2}}$ With a 95% confidence interval (CI) reported prevalence from a similar study had been conducted in the study area 57%) (31) With 5% precision and 10% non-response rate was used. The final sample size for the study was the final sample size with a single population proportion formula is 453. For factors associated with diversified dietary intake the sample size using Epi-info software imperial statistics like odd ration, the proportion of exposed and unexposed with diversified dietary intake and power of 80%, with the ratio of exposed to unexposed 1:1 and 5% level of significance. Finally, the sample size for the second objective, which is calculated for the associated factors For Diversified Dietary intake, is less than the first objective. Therefore, the sample size of the first objective is taken as the final sample size, which is **453.**

### Sampling techniques

From seventeen public health facilities in the Dire Dawa administration stratified by urban and rural strata, by using simple random technique 8 public health facilities (i.e. 4 from urban and 4 from rural) were selected.

The calculated sample size (453) is proportionally allocated to the selected public health facility based on their average number of client flows (client load). Participants in each facility were selected by using a systematic sampling technique after calculating the sampling interval (K) for each facility. To determine whom to be included in our sample we use systematic random sampling then calculate for constant ‘K’ by dividing the total client flow of required public health facilities in 15 days by our required sample size in that facility and the first sample was selected by lottery method between 1 and K, then every K interval value was selected to get required sample size from each public health facility during the required period.

### Data collection tools, techniques, and personnel

Data were collected by interview administered methods using a Structured and pre-tested questionnaires and anthropometric measurement. The quantitative data collection questionnaire has four components: socio-demographic characteristics, Health service factors, and maternal factors. Parts of the questionnaire on the Individual Dietary Diversity Scale (IDDS) were assessed by using a standard questioner developed by Food and Nutrition Technical Assistant (FANTA) [2]. Eight clinical nurses experienced in data collection were recruited as data collectors. The training was given by (PI) to data collectors and supervisors for three days about the objective and methodology of the research on basic skills of anthropometric, interview techniques, data recording, ways of obtaining consent, and on how to maintain confidentiality of information.

### Precautions made to limit exposer to COVID-19 during data collocation

The data collectors were a trend and updated on the current situation of COVID-19 along with precautions that must be implemented during the data collection. The data collectors were provided with personal protective tools (face mask, glove, and sanitizer hand rub) and a proper time and calendar were developed to limit crowding during data collection with other data collectors at the site.

### Variables

#### Dependent variable

Diversified Dietary intake

#### Independent variable

Sociodemographic; residence, family size, monthly income, husband educational level, husband occupation. Health service factors; the number of ANC visits, Counseling in this facility. Maternal factors; experience of illness. Dietary Factor; meal frequency

### Operational definitions and measurements

#### Dietary diversity

Number of individual food groups consumed over 24 hours, inadequate dietary diversity:—When individuals consume less than five food groups, Adequate dietary diversity:—When individuals consume greater than five food groups [2].

#### Meal frequency

Pregnant mothers eating a meal more than 3times per day had adequate meal frequency and less than 3 times inadequate meal frequency [31].

#### Maternal nutritional status

MUAC categorized as undernutrition (MUAC < 23) and normal (MUAC ≥ 23) [32].

### Data processing and data analysis

Data were cleaned and checked by Kobo tool data and it was exported to SPSS version 20 for analysis. Frequency distribution and percentages were computed to describe socio-demographic and other characteristics of respondents and presented in tables and figures. To identify factors associated with Diversified Dietary Intake, binary logistic regression analysis was carried out at two levels, first bivariate logistic regression was performed on each independent variable with the outcome variable, and those variables with a p-value < 0.25 were included in the final model (multivariate analysis). The strength of association was measured using the odds ratio, and 95% confidence intervals. Statistical significance was declared at P-value <0.05. Hosmer and Lemeshow goodness of fit (p-value above) was used to show model fitness in stepwise backward regression analysis. For qualitative data, the narrative data was interpreted and described.

In this analysis, dietary diversity scores were calculated by summing up the number of food groups consumed over 24 hours by the women. The scores greater than or equal to 5 were coded as adequate dietary diversity scores and inadequate dietary diversity otherwise (FHI/FAO/FANTA, 2016) [2]. The women with Mid Upper Arm Circumference (MUAC) less than 23cm were categorized as wasted, based on previous studies.

### Data quality assurance

To maintain consistency, the questionnaire was first translated from English to the local language (Afan-Oromo, somaligna, and Amharic) the native language of the study area, and was back-translated to the English language by professional translators. To evaluate the acceptability and applicability of the procedures and tools a pre-test was administered on 5% of the sample in Gende Gerada health center. Finally, unclear questions were modified before the data collection. To keep completeness and consistency, data collectors were closely supervised before and during the data collection process by the supervisor.

### Ethical considerations

Ethical clearance was obtained from Research Ethical Review Committee of Dire Dawa University, college of medicine and health science. Then, to get the required support, a formal letter was written from the college of medicine and health science to the Dire Dawa administrative health bureau. An informed voluntary, written, and signed consent was obtained from all subjects for their participation after the nature of the study is fully explained to them in their local languages. A thumbprint or signature was used on the consent form. Those who are signed written consent were only participants in the study and confidentiality of response was maintained throughout the research process by giving code for the participant. The entire study participants were informed that data was kept private and confidential and used only for research purposes. The participants were also assured that they have the right to refuse or withdraw if they are not comfortable at any time. Personal privacy and cultural norms were respected.

## Result

### Socio-demographic characteristics

Out of 453 pregnant mothers, 448 of them provided a complete response to the questionnaire marking the response rate of 98.9%. The mean age with a standard deviation of study participants was 27.7 ± 5.01SD and above half of them, 282 (62.9%) were within the age group of 20–29 years. The majority of the participant were urban 289 (64.5%) and educational status, most respondents 124(27.7%) had High and preparatory school. Concerning the respondent’s occupation, 273(60.9%) were housewives. Of the total, 150(33.5%) of husbands education had college and above. Out of the total, 178(39.7%) pregnant mothers monthly income had greater than 3000ETB, 145(32.4%) between 2100-3000ETB **(Table 1).**

**Table 1:** Socio-demographic characteristics of pregnant mothers attending antenatal care at public health facilities in Dire Dawa, Eastern Ethiopia, 2020.

Variable	Frequency	%
**Residence**		
Urban	289	64.5%
Rural	159	35.5%
**Religion**		
Muslim	303	67.6%
Orthodox	93	20.8%
Catholic	12	2.7%
Protestant	38	8.5%
Other	2	0.4%
**Family Size**		
<5	304	67.9%
> = 5	144	32.1%
**Monthly Income**		
<1000	34	7.6%
1100–2000	91	20.3%
2100–3000	145	32.4%
above	178	39.7%
**Husband education level**		
Unable to write and read	78	17.4%
Able to write and read	45	10%
Elementary school	65	14.5%
High and preparatory school	110	24.6%
College and above	150	33.5%
**Husband occupation**		
Daily labor	123	27.5%
Private worker	126	28.1%
Government	127	28.3%
Farmer	72	16.1%
**Age**		
<19	21	4.7%
20–29	285	62.9%
>30	145	32.4%
**Maternal education**		
Unable to write and read	109	24.3%
Able to write and read	43	9.6%
Elementary school	89	19.9%
High and preparatory school	124	27.7%
College and above	83	18.5%
**Maternal occupation**		
housewife	273	60.9%
daily labor	29	6.5%
private worker	75	16.7%
government	48	10.7%
student	23	5.1%

### Maternal and health service factors

Out of 448 participants three fourth 346 (77.2%) of them were no illness in the past four weeks, 160 (35.7%) of respondents there is no counseling about diversified dietary intake, and a majority of 259 (57.8%) of participants reported that they consumed three times meals per day (**Table 2**).

**Table 2:** Maternal and health service factors of a pregnant mother attending antenatal care at public health facilities in Dire Dawa, Eastern Ethiopia, 2020.

Variable	Frequency	Percentage (%)
**Number of meals per day**		
2 times	25	5.6%
3 times	259	57.8%
> = 4 times	164	36.6%
**Counselling About dietary diversity intake?**		
Yes	288	64.3%
No	160	35.7%
**Past four illnesses experienced**		
Yes	102	22.8%
No	346	77.2%

**Fig 1:**
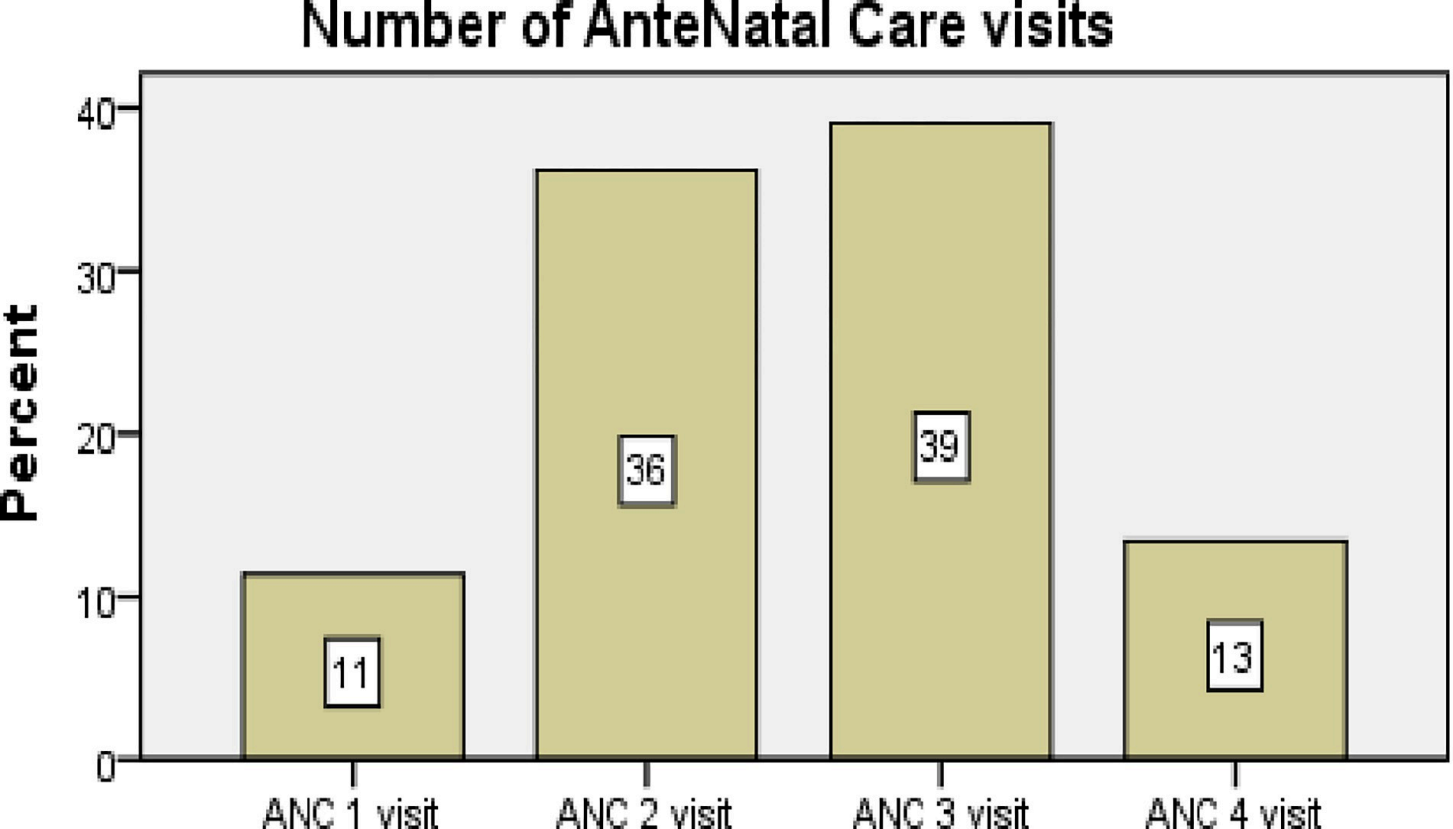
Number of ANC Visit illness for dietary y diversity among pregnant mother attending antenatal care in public health facilities in Dire Dawa, 2020.

While concerning antenatal care practice, 51(11.4%) of a pregnant mother had one ANC visit, 162 (36.2%) had two visits, 175(39.1%) had three visits and 60 (13.4%) of pregnant women had four visit. (**Fig 1**)

### Nutritional status among pregnant mothers

The mean and standard deviation MUAC of the respondent was 25.4cm ±3.5SD cm. Out of the pregnant mothers, 133(29.7%) of them were under nutritional and 315(70.3%) were having normal nutritional status.

### Dietary intake among pregnant mother

**Fig 2:**
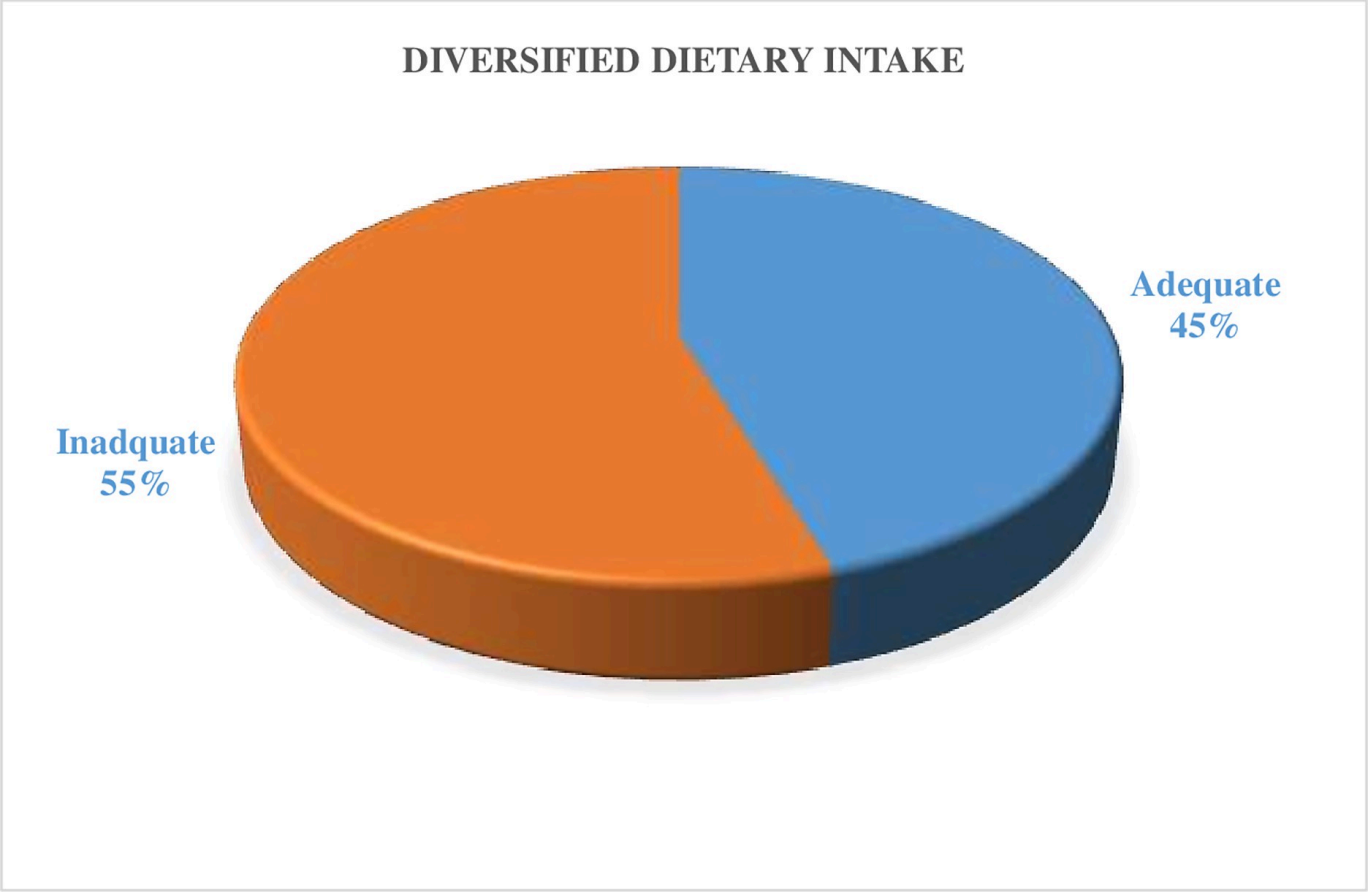
Diversified dietary score of pregnant mother in public health facilities in Dire Dawa, 2020.

**Fig 3:**
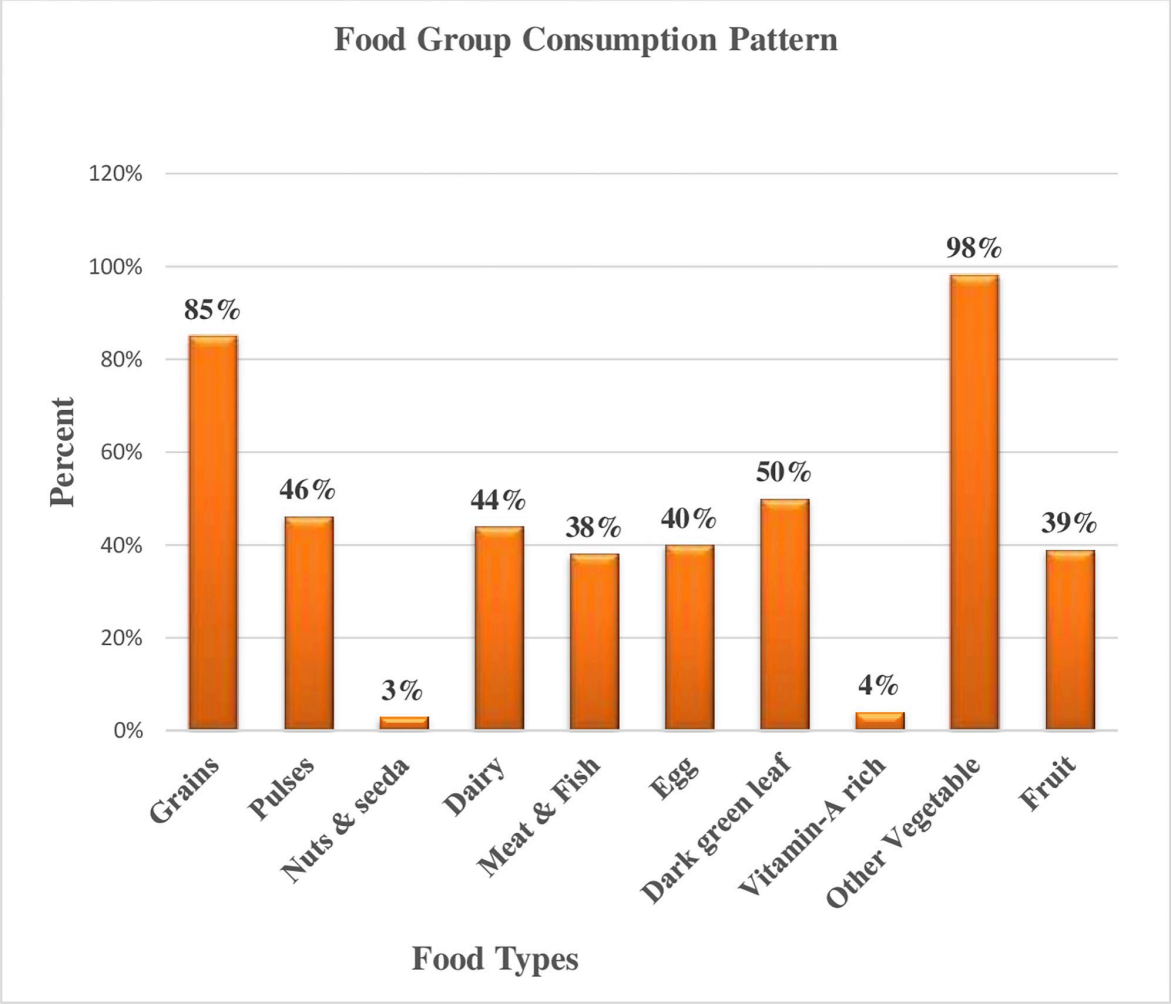
Food groups consumption patterns of pregnant mother in public health facilities in Dire Dawa, 2020.

In this study about 245 (55%); 95%CI: (50, 59.6) pregnant mothers had Inadequate Dietary Diversity and 203(45%); 95%CI: (40.4, 50) pregnant mothers had Adequate Dietary Diversity practice which is recommended by FAO 2016 (> = five food groups) (**Fig 2**). The most commonly consumed food groups were other vegetables 440(98%) followed by grains 381(85%) and dark green leafy vegetables 222(50%) (**Fig 3**).

### Factors associated with diversified dietary intake pregnant mother

The association of outcome variable (Diversified Dietary Intake) and independent variables was assessed using both bivariate and multivariate logistic regression. Accordingly; bivariate analysis revealed that out of fourteen explanatory variables only nine variables such as- educational status, maternal occupation, husband education and occupation, family size, monthly income, meal frequency, information about dietary diversity and MUAC had a statistical association with pregnant mother diversified dietary intake (P-value <0.25) whereas residence, religion, age, past illness of mothers and ANC visit were not significantly associated and excluded from further analysis (**Table 3**).

**Table 3:** Bivariate analysis of factors associated with diversified dietary intake of a pregnant mother attending antenatal care in public health facilities in Dire Dawa, Eastern Ethiopia, 2020.

Variables		Diversified Dietary Intake		COR (95%CI)
		Inadequate (%)	Adequate (%)	
**Resident**				
	Urban	150 (52)	139(48)	0.7(0.5, 1.08)
	Rural	95(60)	64(40)	1
**Religion**				
	Muslim	180(59)	123(41)	1.5(0.09, 23.6)
	orthodox	42(45)	51(55)	0.8(0.05, 13.6)
	catholic	6(50)	6(50)	1.0(0.05, 19.9)
	protestant	16(42)	22(58)	0.7(0.04, 12.5)
	other	1(50)	1(50)	1
**Family size**				
	< 5	151(50)	153(50)	0.5(0.3, 0.8)*
	> = 5	94(65)	50(35)	1
**Monthly income**				
	< 1000	29(85)	5(15)	9.4(3.5, 25.4)*
	1100–2000	66(73)	25(28)	4.3(2.5, 7.4)*
	2100–3000	82(57)	63(43)	2.1(1.3, 3.3)*
	above	68(38)	110(62)	1
**Husband educational level**				
	Unable to write and read	57(73)	21(27)	5.3(2.8, 9.6)*
	Able to write and read	27(60)	18(40)	2.9(1.5, 5.8)*
	Elementary school	46(71)	19(29)	4.7(2.5, 8.8)*
	High and preparatory school	64(58)	46(42)	2.7(1.6, 4.5)*
	College and above	51(34)	99(66)	1
**Husband occupation**				
	daily labor	92(75)	31(25)	4.8(2.8, 8.4)*
	farmer	45(63)	27(38)	2.7(1.5, 4.9)*
	private worker	60(48)	66(52)	1.5(0.9, 2.5)
	government	48(39)	79(62)	1
**Age of mother**				
	<19	13(62)	8(38)	1.1(0.4, 2.9)
	20–29	147(51)	135(48)	0.8 (0.5, 1.2)
	>30	85(59)	160(41)	1
**Maternal educational level**				
	Unable to write and read	75(69)	34(31)	6.1(3.2, 11.5)*
	Able to write and read	26(60)	17(40)	4.2(1.9, 9.3)*
	Elementary school	65(73)	24(27)	7.5(3.8, 14.8)*
	High and preparatory school	57(46)	67(54)	2.4(1.3, 4.3)*
	College and above	22(26)	61(74)	1
**Occupation of mother**				
	housewife	170(62)	103(38)	3.01(1.6, 5.7)*
	student	11(48)	12(52)	1.7(0.6, 4.6)
	daily labor	19(65)	10(35)	3.5 (1.3, 9.1)*
	private worker	28(37)	47(63)	1.08(0.5, 2.3)
	government	17(35)	31(65)	1
**Number of meals per day**				
	2 times	24(96)	1(4)	38.3 (5.1, 290.5)*
	3 times	159(61)	103(39)	2.5 (1.6, 3.7)*
	> = 4 times	62(39)	99(61)	1
**Past four weeks of illness**				
	Yes	51(50)	51(50)	0.8 (0.5, 1.2)
	No	194(56)	152(44)	1
**Number of Ante Natal Care visits**				
	1^st^visit	30(59)	21(41)	1.6 (0.7, 3.5)
	2^nd^visit	90(56)	72(44)	1.4 (0.8, 2.6)
	3^rd^visit	97(54)	78(45)	1.4(0.8, 2.6)
	4^th^visit	28(45)	32(53)	1
**Counseling**				
	No	98(61)	62(39)	1.5 (1.02, 2.24)*
	Yes	147(51)	141(49)	1
**MUAC**				
	< 23cm	87(65)	46(35)	1.9(1.2, 2.8)*
	≥ 23cm	158(50)	157(50)	1

*P<0.25 *CI*, *Confidence Interval*, *COR*, *Crude Odds Ratio*.

After bivariate analysis; those variables showing significant association were entered into multivariate logistic regression in order to rule out the effect of confounding variables. As a result four of contributing factors such as, Household monthly income, maternal education, information about dietary diversity, and meal frequency were the variables that were significantly associated with diversified dietary intake (P-value <0.05).

In this study, pregnant Mothers who had elementary education were almost 3.8 times more likely to have inadequate dietary diversity as compared to those who had college and above education (AOR = 3.8, 95% CI:1.5, 9.9). Concerning family monthly income; those who had an estimated monthly income of less than 1000ETB had 4.4 times more likely an increase the chance of inadequate dietary diversity as compared to those who had an estimated monthly income of greater than 3000ETB (AOR = 4.4, 95% CI: 1.3, 14.6) and also those who had an estimated monthly income of 1100-2000ETTB increase the likelihood of attaining inadequate dietary diversity by 2.7 times as compared to those who had an estimated monthly income greater than 3000ETB (AOR = 2.7, 95% CI:1.3, 5.5).

This study also showed that pregnant mothers who had not exposure of dietary diversity information were 2.2 times more likely having inadequate dietary diversity as compared to those who were the exposure of dietary diversity information (AOR = 2.2, 95% CI:1.1, 2.8). Furthermore; those pregnant mothers who had two meals per day were 16.6 times more likely to have inadequate dietary diversity as compared to those who had greater than four meals per day (AOR = 16.6, 95% CI:2.04,135.8) (**Table 4**).

**Table 4:** Multivariate analysis of factors associated with diversified dietary intake of a pregnant mother attending antenatal care in public health facilities in Dire Dawa, Eastern Ethiopia, 2020.

	Diversified Dietary Intake			
Variables	Inadequate (%)	Adequate (%)	COR (95%CI)	AOR (95% CI)
**Monthly income**				
< 1000	29(85)	5(15)	9.4(3.5, 25.4)*	4.4 (1.3, 14.6)*
1100–2000	66(73)	25(28)	4.3(2.5, 7.4)*	2.7 (1.3, 5.5)*
2100–3000	82(57)	63(43)	2.1(1.3, 3.3)*	1.3(0.8, 2.2)
above	68(38)	110(62)	1	1
**Maternal educational level**				
Unable to write/read	75(69)	34(31)	6.1(3.2, 11.5)*	2.04(0.61, 6.81)
Able to write and read	26(60)	17(40)	4.2(1.9, 9.3)*	1.9(0.6, 6.05)
Elementary school	65(73)	24(27)	7.5(3.8, 14.8)*	3.8(1.5, 9.9)**
High / preparatory	57(46)	67(54)	2.4(1.3, 4.3)*	1.8(0.8, 4.01)
College and above	22(26)	61(74)	1	1
**Number of meals per day**				
2 times	24(96)	1(4)	38.3 (5.1, 290.5)*	16.6(2.04,135.8)**
3 times	159(61)	103(39)	2.5 (1.6, 3.7)*	1.5(0.92, 2.4)
> = 4 times	62(39)	99(61)	1	1
**Counseling**				
No	98(61)	62(39)	1.5 (1.02, 2.24)*	2.2(1.1, 2.8)*
Yes	147(51)	141(49)	1	1

* P value<0.05 &

**P value <0.01

AOR, Adjusted Odds Ratio, CI, Confidence Interval, COR, Crude Odds Ratio

## Discussion

This study found that about 55% of a pregnant mothers were inadequate dietary diversity (< 5 food groups) while 45% of them were adequate dietary diversity (> = 5 food groups). The finding of this study was lower as compared to the inadequate dietary diversity in which pregnant mothers should consume 57% study done in Dire Dawa 2016, [30], 59.6% in Gambela [33], 73% in Mekele [34], and 56.4% in Axum Northern Ethiopia [35]. And also this finding was higher as compared to inadequate dietary diversity in which pregnant mothers should consume study conducted in Pakistan 2014, 11% [22], 25.6% in Nepal 2016 [36], 37% in Bangladesh 2015 [37], 22.7% in Malaysia 2015 [38], 39% in Laikipia, Kenya 2016 [39], 25.4% in Shashemene 2017 [40] and 38.8% in Alamata 2017 [31]. The possible reason for this great discrepancy may be due to differences with respect to socio-demographic, socio-economic health characteristics of the population, and sample size variation. Many studies also used the nine or fourteen food groups, and those consumed four or more food groups of the fourteen food groups were considered as they achieved diversified dietary intake which result from high proportion of diet diversity.

When comparing individual food group consumption patterns, grain, other vegetables, and dark green leafy vegetables are the commonest consumption in the study area due to being easily available and cheapest for other food groups. In the particular conception of the animal sources of food such as dairy products, egg, meat, and fish were observed among some number of participants. This is also because of seasonal variability since a dairy products like milk and milk products are accessible in the study area and the practice of eating meat and fish was mostly oriented towards the celebration of religious holidays, marriage ceremony and other social welfares.

Maternal education, Monthly income, Meal frequency, and information about dietary diversity are significantly associated with dietary diversity intake.

Pregnant mothers who had elementary education were almost 4 times more likely to have inadequate dietary diversity as compared to those who had college and above education. This is in line with a study done in rural Bangladesh 2015 [37], southwest Bangladesh 2016 [41], Bangladesh 2013 [42], Kenya 2016 [39], Alamata [31], and Hossana, Ethiopia, [43], Tigray Ethiopia [44]. The reason behind here is because the most educated pregnant mother had good food choices due to knowledge about the importance of food and were more likely to understand educational messages transfer through different media channels and also a pregnant mothers who had education have greater awareness about how to utilize available resources for the improvement of their diet quality.

Family income was significantly associated with pregnant mother dietary diversity. As a result, the study revealed those who had a monthly income of less than 1000ETB had 4.4 times more likely an increase in the chance of inadequate dietary diversity as compared to those who had a monthly income of greater than 3000 ETB. This result is supported by different studies conducted in India [45], Ahvaz-Iran [7], Shashemene 2017 [40]. As income levels decreased, the chance of pregnant mothers being inadequate in dietary diversity was high [46]. The possible reason behind here is income and the prices of foods determine food access. Mothers with higher income have the purchase power to afford foods from the market even not available at home.

In this study, pregnant mothers who had two meals per day was 16.6 times more likely to have inadequate dietary diversity as compared to those who had greater than four meals per day. This finding is in line with the other studies conducted in Hossana [43], Alamata [31], and Bale, Ethiopia, [47]. This might be related to their opportunity of having greater than two meals may have the chance of consuming different categories of food group.

This study also showed that pregnant mothers who had not the exposure of dietary diversity information were 2.2 times more likely to have inadequate dietary diversity as compared to those who were the exposure to dietary diversity information. This finding is in line with studies carried out Hossana town south Ethiopia [48], in Jijiga town [49], in Guto Gida Woreda, East Wollega, and Central Gonder [50, 51]. This is since those who had exposure to dietary diversity have better knowledge about the importance of a diversified diets to maintain proper health and pregnancy outcomes.

This study may have certain limitations like due to the cross-sectional design this study cannot make the cause and effect relationship between different factors with the outcome variable. Besides, this study might not give the exact figure of the dietary diversity practice due to recall bias so the 24-hour dietary recall may not truly represent the usual intake and is also unable to generalize to the population because of the health-facility nature of this study.

## Conclusions

This study suggested that around 55% of respondents had inadequate dietary diversity mainly defined by the inclusion of ten food groups. The study showed that pregnant mothers’ educational status, family income, meal frequency, and information about dietary diversity have contributed to low diversified dietary intake. There is a need for enhancing dietary diversity or food groups and promotion of awareness on nutritional benefits especially consumption of at least five food groups for pregnant women. Increase meal frequency and health service utilization. And also support health facilities to train their health professionals especially, health extension workers should provide regular advice to women about the nutritional value of consuming different food groups.

## Supporting information

S1 Text Questionnaire used to assess diversified dietary intake and associated factors among pregnant mothers. (DOCX)

S1 Data Raw data used in the analysis of diversified dietary intake and associated factors among pregnant mothers. (SAV)
