# Supplementary material for: Diversified dietary intake and associated factors among pregnant mothers attending antenatal care follow-up in public health facilities of Dire Dawa, Eastern Ethiopia
Source: PLOS Glob Public Health. 2022 Jun 30;2(6):e0000002. doi: 10.1371/journal.pgph.0000002 (PMC10022029; doi:10.1371/journal.pgph.0000002)
Supplement: S1 Text — (DOCX) [file pgph.0000002.s001.docx]

**Questionnaire used to assess diversified dietary intake and associated factors among Pregnant Mothers**

**English Questioner**

**Part I: Sociodemographic Characteristics**

| **No** | **Question** | **Category** | **Code** |
| --- | --- | --- | --- |
| Q101 | Place of residence | 1. urban  2. rural |  |
| Q102 | Religion | 1.muslim  2.orthodox  3.catholic  4.protestant  5.other__________ |  |
| Q103 | Family size | 1, <5  2, ≥5 |  |
| Q104 | Monthly income | 1. < 1000  2. 1100-2000  3. 2100-3000  4. above |  |
| Q105 | Husband educational level | 1. Unable to write and read  2. Able to write and read  3. Elementary school  4. High and preparatory school  5. College and above |  |
| Q106 | Husband occupation | 1.daily labor  2.private worker  3.government  4. farmer |  |
| Q107 | Age of mother |  |  |
| Q108 | Maternal educational level | 1. Unable to write and read  2. Able to write and read  3. Elementary school  4. High and preparatory school  5. College and above |  |
| Q109 | Occupation of mother | 1.housewife  2.daily labor  3.private worker  4.government  5. student |  |

**Part II: Maternal factors and Health service factors**

| **No** | **Question** | **Category** | **Code** |
| --- | --- | --- | --- |
| Q201 | Number of meal per day |  |  |
| Q202 | Past four weeks of illness | 1. Yes 2. No |  |
| Q203 | Number of AnteNatal Care visits |  |  |
| Q204 | Is there any counseling in this facility about dietary diversity intake? | 1. Yes 2. No |  |

**Part III: Maternal** **Consumption of Food Groups**

| No | Food group | 1.yes 2.no |
| --- | --- | --- |
| 301 | corn/maize, rice, wheat, sorghum, millet or any other grains or foods made from these (e.g. bread, noodles, porridge or other grain products) |  |
| 302 | white potatoes, white yam, white cassava, or other foods made from roots |  |
| 303 | pumpkin, carrot, squash, or sweet potato that are orange inside + another locally available vitamin A-rich vegetables (e.g. red sweet pepper) |  |
| 304 | dark green leafy vegetables, including wild forms + locally available vitamin A rich leaves such as amaranth, cassava leaves, kale, spinach |  |
| 305 | other vegetables (e.g. tomato, onion, eggplant) |  |
| 306 | ripe mango, cantaloupe, apricot (fresh or dried), ripe papaya, dried peach, and 100% fruit juice made from these |  |
| 307 | other fruits, including wild fruits and 100% fruit juice made from these |  |
| 308 | Any liver, kidney, heart or other organ meats or blood-based foods |  |
| 309 | Any beef, pork, lamb, goat, rabbit, game, chicken, duck, other birds, insects |  |
| 310 | Any eggs from chicken, duck, guinea fowl or any other egg |  |
| 311 | Any fresh or dried fish or shellfish |  |
| 312 | dried beans, dried peas, lentils, nuts, seeds or foods made from these (eg. hummus, peanut butter) |  |
| 313 | milk, cheese, yogurt or other milk products |  |
| 314 | oil, fats or butter added to food or used for cooking |  |
| 315 | sugar, honey, sweetened soda or sweetened juice drinks, sugary foods such as chocolates, candies, cookies, and cakes |  |
| 316 | spices (black pepper, salt), condiments (soy sauce, hot sauce), coffee, tea, alcoholic beverages |  |

**Anthropometric**

Mid Upper Arm Circumference (MUAC)
